# Quantitative Single-Molecule Localization Microscopy

**DOI:** 10.1146/annurev-biophys-111622-091212

**Published:** 2023-05-09

**Authors:** Siewert Hugelier, P.L. Colosi, Melike Lakadamyali

**Affiliations:** 1 Department of Physiology, Perelman School of Medicine, University of Pennsylvania, Philadelphia, Pennsylvania, USA; 2 Department of Cell and Developmental Biology, Perelman School of Medicine, University of Pennsylvania, Philadelphia, Pennsylvania, USA; 3 Epigenetics Institute, Perelman School of Medicine, University of Pennsylvania, Philadelphia, Pennsylvania, USA

**Keywords:** quantitative analysis, super-resolution nanoscopy, single-molecule localization microscopy, quantitative cell biology

## Abstract

Super-resolution fluorescence microscopy allows the investigation of cellular structures at nanoscale resolution using light. Current developments in super-resolution microscopy have focused on reliable quantification of the underlying biological data. In this review, we first describe the basic principles of super-resolution microscopy techniques such as stimulated emission depletion (STED) microscopy and single-molecule localization microscopy (SMLM), and then give a broad overview of methodological developments to quantify super-resolution data, particularly those geared toward SMLM data. We cover commonly used techniques such as spatial point pattern analysis, colocalization, and protein copy number quantification but also describe more advanced techniques such as structural modeling, single-particle tracking, and biosensing. Finally, we provide an outlook on exciting new research directions to which quantitative super-resolution microscopy might be applied.

## INTRODUCTION

Fluorescence microscopy is a powerful tool that offers several advantages over other microscopy methods including electron microscopy. Light is noninvasive, and fluorescence tagging of the biological entity of interest provides high contrast and molecular specificity. While light microscopy began as a descriptive and qualitative tool, it has evolved into a highly quantitative method in biological studies. For example, fluorescence correlation spectroscopy (FCS) allows measurements of concentrations of molecules inside living cells (42), fluorescence resonance energy transfer (FRET) enables quantification of molecular interactions or conformational changes within individual molecules (1), and single-step photobleaching has been used extensively to determine the subunit stoichiometry of sparsely expressed small protein complexes (39, 40). However, until the 2000s, a major limitation of fluorescence light microscopy was its low spatial resolution (approximately 250 nm in *x-y* and approximately 500 nm in *z*) due to diffraction, meaning that light microscopy could only be applied to bulk quantification of many molecules residing within this diffraction-limited volume or to sparsely expressed or labeled molecules that did not overlap within the diffraction-limited volume. Thus, the development of far-field super-resolution light microscopy, which overcame this limitation, revolutionized the study of subcellular biology.

The super-resolution revolution began with the development of stimulated emission depletion (STED) microscopy (87, 88), followed by stochastic optical reconstruction microscopy (STORM) (140); (fluorescence) photoactivated localization microscopy (f/PALM) (15, 64); and, later, DNA-point accumulation in nanoscale topography (PAINT) (82, 152), an evolution of the original PAINT method by Sharonov & Hochstrasser (159). The latter three methods are now collectively referred to as single-molecule localization microscopy (SMLM) (92). The early days of super-resolution microscopy focused largely on proof-of-concept demonstrations and technological advances such as extending the methods to 3D and multicolor imaging (12, 73, 149). As the techniques have matured, they have become important discovery tools in cell biology and as such have needed to become more quantitative. Recent years have seen an explosion of analysis algorithms to improve both the preprocessing and postprocessing of super-resolution data, which enabled in situ structural biology with light microscopy, measurement of molecular clustering, and determination of protein copy number within molecular clusters, among other advances. In this review, we focus on the advances in quantitative super-resolution light microscopy, with a particular emphasis on quantitative SMLM.

## OVERVIEW OF SUPER-RESOLUTION METHODS

### Single-Molecule Localization Microscopy

**Figure 1:**
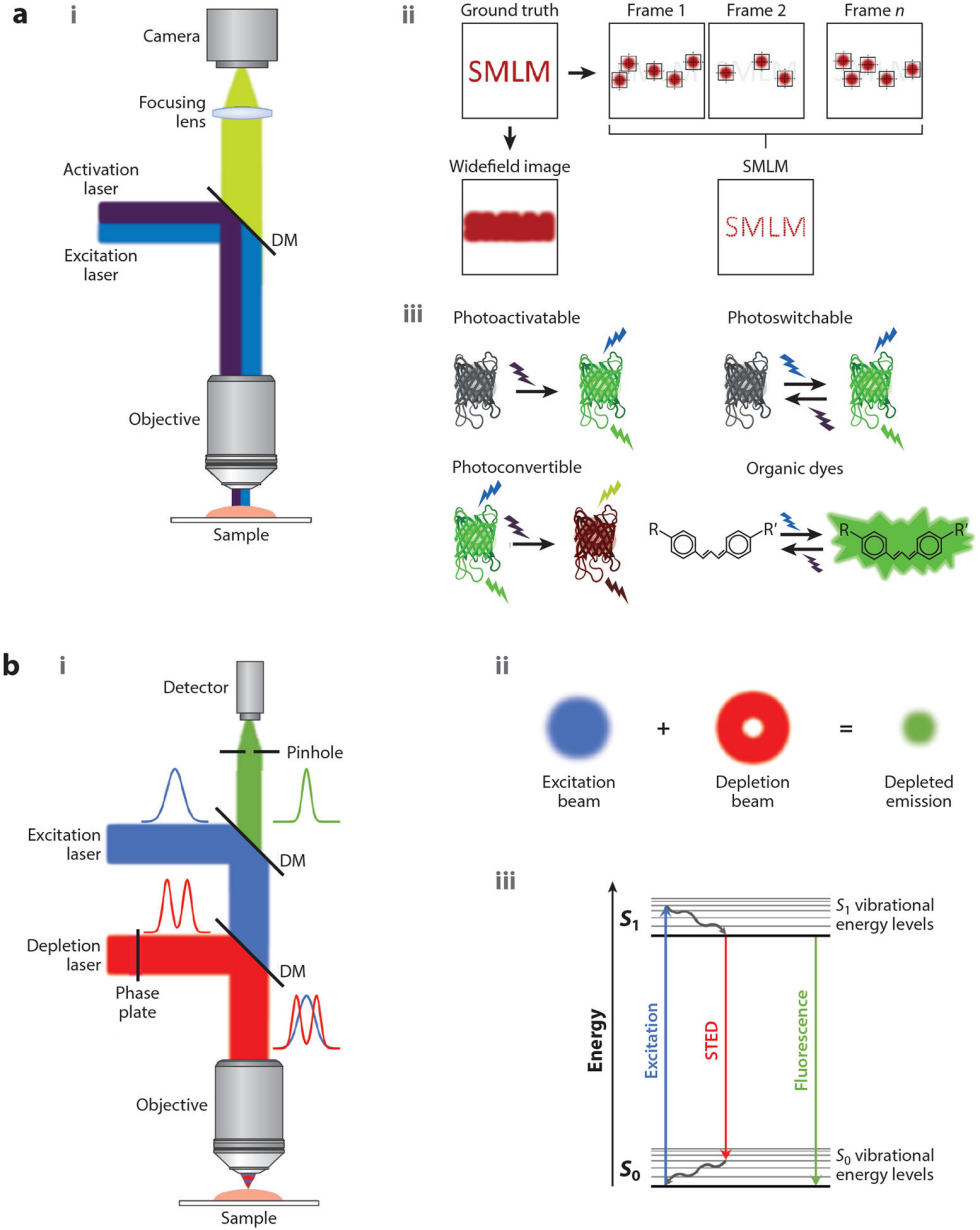
Schematic representation of the microscope modalities covered in this review. (*a*) SMLM. The microscope setup (*i*), the working principle (*ii*), and some examples to achieve *on/off* switching of fluorescent proteins and organic dyes (*iii*) are shown. (*b*) STED microscopy. The microscope setup (*i*), the working principle (*ii*), and a Jablonski diagram of the STED excitation and emission (*iii*) are shown. Abbreviations: DM, dichroic mirror; SMLM, single-molecule localization microscopy; STED, stimulated emission depletion.

STORM, f/PALM, and DNA-PAINT belong to a subclass of super-resolution microscopy methods that are collectively referred to as SMLM (see Figure 1a, i) as they all share the same concept for improving spatial resolution. The working principle behind these methods has been extensively reviewed (92, 146); thus, we provide only a brief description and instead focus on quantitative aspects of SMLM. In SMLM, the biological entity of interest is labeled with probes that can cycle between *on* and *off* states or that can change their spectral properties (e.g., switch from green to red) (Figure 1a, iii). In STORM and f/PALM, the *on/off* switching or the photoconversion is achieved by fluorophores whose photophysical properties can be tuned with light illumination and often with the use of chemical buffers. In DNA-PAINT, the *on/off* switching is achieved via the binding and unbinding of fluorophore-labeled single-stranded oligonucleotides (imager oligos) to and from their target oligonucleotides (docking oligos). In all cases, the *on/off* switching or the photoconversion allows control over the proportion of fluorophores that are in the *on* state at any given time. Sparsely activating, immobilizing, or photoconverting a small proportion of fluorophores at a time ensures that their images do not overlap within a diffraction-limited volume (Figure 1a, ii). The position of each fluorophore can then be determined with high precision (tens of nanometers) by fitting its image to a mathematical function (often a Gaussian). Through iterative cycles of fluorophore activation and localization, it is possible to reconstruct a pointillistic, high-resolution image of the underlying structure reaching a spatial resolution that is one order of magnitude better than the diffraction limit. The pointillistic nature of the SMLM images, as well as the often-stochastic nature of the photoswitching behavior, poses special challenges for image quantification, which are further discussed below.

### Stimulated Emission Depletion

The working principle of STED (see Figure 1b, i) has also been reviewed extensively (144, 177). In brief, molecules within a diffraction-limited volume are excited using the excitation beam. A donut-shaped depletion (or STED) beam forces the molecules where the STED beam intensity is high back to the ground state through stimulated emission such that the fluorescence signal from these molecules is suppressed (Figure 1b, ii, iii). This geometry has the effect of shrinking the excitation volume to only those molecules that are at the center of the depletion beam, where the STED laser intensity is zero. Stimulated depletion is only one mechanism for switching *off* molecules in the donut area. Photoswitchable molecules can also be used in a related method known as reversible saturable optical fluorescence transitions (RESOLFT) (61, 67).

More recently, STED has been combined with the concept of localization in a new method called minimal photon fluxes (MINFLUX) (11, 59) and the related methodology MINSTED (183). In MINFLUX, a donut-shaped probing excitation beam is used to localize photoswitchable molecules by iteratively scanning the beam around the molecule to improve the precision of localization to 1–3 nm. Related methods such as SIMFLUX (28), repetitive optical selective exposure (57, 58), and modulated localization (80) combine structured illumination with the concept of fluorophore localization to achieve high-precision localization in a larger field of view than MINFLUX and MINSTED, thereby improving throughput (18).

Methods like STED and RESOLFT require minimal preprocessing (often, images are only deconvolved to improve resolution further). However, the typical image postprocessing tools that have been developed for intensity-based images can be applied to these images, since they are intrinsically intensity based rather than point cloud based. We highlight some advanced quantitative extensions of these methods, such as STED-FCS and single-particle tracking (SPT) with MINFLUX, but our focus centers largely on quantification of SMLM images.

## SINGLE-MOLECULE LOCALIZATION MICROSCOPY IMAGE ANALYSIS

### Preprocessing of Single-Molecule Localization Microscopy Data

To obtain robust quantitative super-resolution data, a variety of criteria must be considered, including optimized sample preparation, use of appropriate (imaging) buffers, and correct microscope settings (e.g., dichroic mirrors, laser power and wavelength). Assuming that all imaging precautions have been taken (see 173), a crucial part of quantitative SMLM is unbiased localization of the fluorophores, which includes steps like background removal (structured or unstructured background) (70, 74, 109, 113), photobleaching correction (75, 128, 135, 176), drift correction (10, 27, 55, 112, 186), and 2D and 3D emitter detection and localization (9, 117, 125, 127, 163). Many of the works on these topics contain open-source algorithms that can be used within platforms such as ImageJ/FIJI, MATLAB, or Python. Moreover, to make the quantitative analysis of the super-resolution data from different microscope modalities accessible, several efforts have been made to create a platform that combines these different steps of the analysis pipeline (33, 102, 107, 115, 134).

An important consideration in the development of quantitative algorithms is that they should be properly evaluated and compared to other methods. In some cases, it is possible to quantify fundamental limits to the performance of the algorithm, such as the Cramer-Rao lower bound for the 3D localization precision (56, 121). However, in most cases, this quantification requires data for which the ground truth is known. Ground truth information may be experimentally obtained [e.g., by using well-defined DNA-origami structures (81, 150)], but the gold standard is using simulated images. Super-resolution images can be readily simulated and tailored to the applications at hand [e.g., point spread function (PSF) engineering, camera type, structured background], and a wide range of open-source tools is available to do this (48, 119, 142, 172, 175). With the rise of open-source tools and software, a reproducible and more quantitative evaluation of the different methods is also needed. A first effort to compare different metrics (e.g., accuracy, precision, speed, emitter density) was performed by Sage et al. (142) and later updated to include more state-of-the-art techniques (143). These efforts have allowed direct comparison of different methods to make it easier to choose which algorithm to use for specific applications (leaderboards are continuously updated and can be consulted at https://srm.epfl.ch/Challenge).

In recent years, methods that investigate the reliability and confidence of the imaging data have been developed as well (30, 106, 110, 114). These methods of quantification can be used throughout the imaging process to assess the quality of the data to maximize accuracy and resolution and to benefit the downstream analysis. For example, in the case of the Haar wavelet kernel analysis (HAWK) (106), the super-resolved image is compared to a HAWK-processed reconstruction reference image to map and quantify structural differences between them. This procedure allows for estimating image quality, reliability, and artifact detection without the need to use intensity information, which is a common drawback of other methods.

### Statistical Analysis of Single-Molecule Localization Microscopy Data

**Figure 2:**
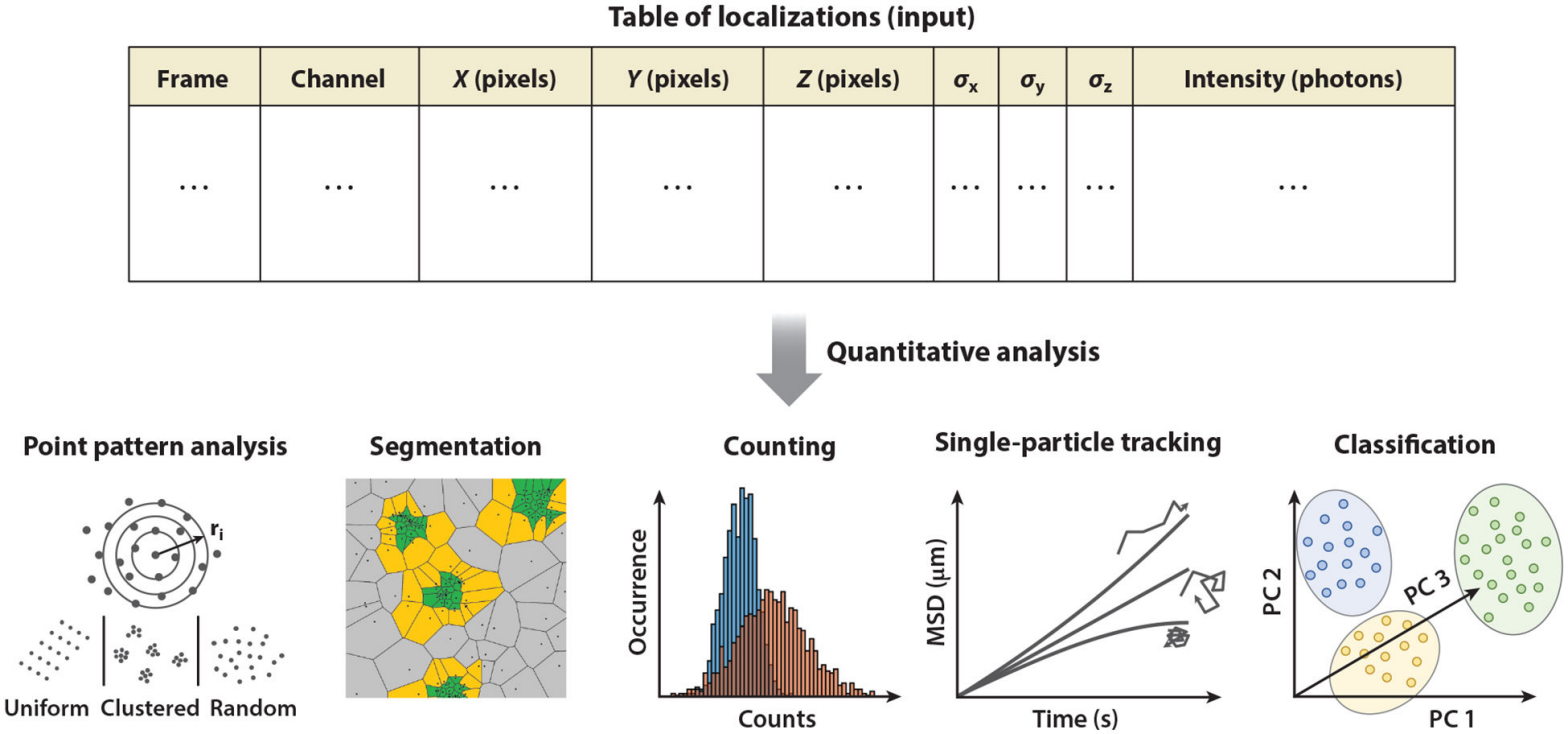
A broad range of quantitative analysis techniques that use input localizations (probe positions) to quantify different applications in cell biology. Abbreviations: MSD, mean square displacement; PC, principal component.

In quantitative super-resolution microscopy, a significant portion of the conclusions are related to the resolution, quality, and spatial or structural details revealed in the image. Statistical approaches are therefore important to quantify these properties (Figure 2). For example, the image resolution is dependent on probe properties (e.g., detected photons), imaging properties (e.g., pixel size), and sample properties (e.g., labeling density) (35). Some of these considerations can theoretically be described based on statistical theorems, such as the Nyquist-Shannon sampling theorem for labeling density (45, 120, 158) or the analytical expression for localization precision (165), but in practice, other factors (e.g., probe size) also limit resolution. Therefore, to more accurately quantify the spatial resolution in experimental images, several analysis methods have been developed, such as Fourier ring correlation (89, 118) and parameter-free estimation of image resolution (38). As an example, the parameter-free method (38) calculates the correlation of a single image in the frequency domain with respect to a frequency mask of decreasing size. This method takes into account that high-frequency information is related to noise, and low-frequency information is related to structural information. This means that, at first, there will be a steady increase in correlation when the noise is removed; after reaching a maximum, the correlation will decrease again as structural information is removed. Therefore, the image resolution is determined by determining the point where correlation is maximal.

Several postprocessing algorithms also make use of statistical analysis. Pair-correlation functions, for example, have been used to quantify true spatial organization by addressing imaging artifacts such as overcounting due to multiple blinking (see the section titled Protein Copy Number Quantification in Single-Molecule Localization Microscopy) (155, 174), to quantify colocalization (see the section titled Colocalization) (103, 153, 166), to perform drift correction (27, 112), and to align images (65, 66, 153). In the image alignment application, pair-correlations are used as a way of performing model-free particle averaging to increase the signal-to-noise ratio and, thus, the effective image resolution (see the section titled Structural Biology and Structural Modeling). Other statistical methods also play an important role in the spatial point analysis of data clusters. A well-known example is the Ripley’s K function and its derivatives (86, 136), which have been used in both 2D and 3D imaging applications obtained with different microscopy modalities (3, 54, 91, 102, 130, 131). These functions use second-moment properties to describe the relationship between the clusters present in a sample by comparing the number of found points within a given distance to what would be expected if there was complete spatial randomness. Deviations from complete spatial randomness then indicate scales of clustering and/or dispersion.

### Clustering and Segmentation of Single-Molecule Localization Microscopy Data

Statistical analyses such as Ripley’s K function, discussed above, are useful for extracting information on the average distribution of points in the SMLM images. However, they do not provide information on individual clusters and structures or their heterogeneity. Clustering and segmentation methods (Figure 2) group SMLM localizations into discrete objects (clusters) that represent collections of molecules or structures of interest. Thus, these methods allow extraction of properties of individual objects and characterization of heterogeneity in the SMLM data.

#### Density-based methods.

Density-based spatial clustering of applications with noise (DBSCAN) can segment clusters of arbitrary shape and only requires a small number of user-defined parameters, which makes it a popular method for clustering SMLM data. The algorithm groups points with many nearby neighbors as clusters and marks any points that have neighbors too far away as noise. Many studies have used DBSCAN to segment SMLM data in diverse biological settings (91, 102, 126). However, one disadvantage of this method is that parameter selection is subjective, as it requires two input parameters, *ε*, the neighborhood radius, and *MinPts,* the minimum number of localizations within *ε* required to be considered a cluster. It is also relatively slow in comparison to more recent density-based algorithms (111), especially as data sets get larger.

#### Voronoi tessellation–based methods.

Voronoi tessellation is a mathematical concept that has been adopted for use in SMLM. This method clusters point cloud data by dividing the space around each SMLM localization into polygonal regions (Voronoi cells) defined by Euclidean distances to their nearest neighbors (4, 19, 94). Each Voronoi cell is centered around one localization and is constructed by edges (lines in this 2D application) that are equidistant to the two nearest sites (i.e., localizations). The cell areas or other cell parameters are then used as thresholds for segmenting the localizations into clusters. For example, regions of the Voronoi diagram with smaller cell areas translate to regions with higher molecular density. Thus, high-density regions can be segmented by placing a threshold on the Voronoi cell area and selecting cells that are smaller than the threshold. Cells that fulfill this criterion and are also neighbors of one another can get grouped together to form a clustered object (94). Thresholds can be set manually or automatically based on comparison between Voronoi cell attributes and a reference distribution (e.g., complete spatial randomness or uniform distribution) (4, 94). Voronoi tessellation has been adapted for the clustering of 3D SMLM data (4) and has been useful for scalable clustering (e.g., from individual nanoclusters up to larger, more complex cellular structures) in several biological applications (60, 62, 122, 130).

#### Bayesian clustering methods.

Bayesian methods seek to control for uncertainties in molecule localization, background signal, and user bias by employing algorithms that propose many potential clustering schematics based on the data being analyzed and scoring those schematics according to a Bayesian generative model (53). In the approach of Griffié et al. (53), every localization in a region of interest (ROI) is assumed to be a molecule whose coordinates come with errors because of the localization process, and the center of each potential cluster and the radii of the clusters are assumed to be distributed uniformly over the ROI. Localizations are then assigned either as background or as part of the cluster. This cluster configuration is then compared to the Bayesian generative model (which assumes Gaussian clusters and a completely spatially random background) and given a posterior probability. Thousands of potential configurations are investigated in this way, and the best-scoring configuration is then the output of the algorithm. Bayesian methods have also been adapted for clustering of 3D SMLM data, in addition to that of 2D data (54).

While the reduction of user input may be helpful for reducing bias, assumptions about what a localization truly represents can become problematic. Localizations that are assumed to have come from separate molecules (rather than one molecule that has blinked multiple times) by an algorithm could output a proposal with artificially large or wholly false clusters. In fact, for all of the clustering algorithms described above, careful attention to the reduction of blinking artifacts is important.

### Protein Copy Number Quantification in Single-Molecule Localization Microscopy

SMLM is particularly useful for studying the characteristics of protein distribution (24, 181) and has been used to identify protein nanodomains, nanoscale areas of a cell wherein certain proteins cluster together (Figure 2). Prominent examples include lipid rafts (162) and receptor clustering (31, 76, 97, 145, 154). In the time since these nanodomains were described, questions have arisen about the true number of proteins making up such clusters, as artifacts intrinsic to the SMLM labeling and the data acquisition process make interpretation of clustered SMLM data less straightforward (5, 13, 37). In SMLM imaging modalities like STORM and PALM, undercounting of localizations can come from incomplete labeling, fluorophore failure to photoactivate, and premature photobleaching, leading to artificially sparse reconstruction of structures. In contrast, overcounting from repeated captures of the same fluorophore across many frames makes for artificially dense reconstructions. Both scenarios are problematic for later clustering and segmentation steps in the data analysis process and for biological interpretation of the reconstructed image.

Initial methods to address these issues focused on the problem of multiple fluorophore blinks. These methods included simple time thresholds in which localizations that appeared within a time shorter than the threshold were combined together (5, 6, 29). In addition, kinetic models of fluorophore photophysics have been developed to account for fluorophore blinking (137). However, these methods either are too simplistic and fail in high-density labeling scenarios (time threshold) or do not fully capture the complexity of fluorophore photophysics (kinetic models). More recently, several advanced methods have been developed to account for blinking. These include pairwise distance distribution correction, which uses pairwise distance distributions of molecules separated temporally by several frames longer than the lifetime of a fluorophore to determine a true pairwise distance distribution (17). In addition to these computational methods, an experimental method for addressing the stochastic nature of fluorophore photophysics in STORM or PALM is the use of quantitative DNA-PAINT, also known as qPAINT (81). In qPAINT, bright and dark times of fluorophores are tied to well-defined kinetic constants of DNA binding and the influx rate of imager strands, rather than the stochasticity of dye photophysics. Thus, well-characterized blinking translates to protein counting when the frequency of blinks in a certain time period from a single protein labeled with a single docking strand is determined. If the frequency for one protein is four blinks within a given time period, then a cluster of four proteins will blink with four times the frequency (given that the rate of influx of imaging strands remains constant).

While these approaches can account for artifacts related to fluorophore blinking, they do not account for other artifacts coming from failed localizations or unknown labeling stoichiometry. In the sections below, we describe techniques that account for these additional artifacts to properly determine protein clustering and protein copy number.

#### Titration methods.

Titration methods utilize careful modulation of labeling density by, for example, titrating the concentration of the fluorescent antibody (13, 41, 60). Generally, the number of SMLM localizations associated with a single secondary antibody is found by diluting the antibody concentration in a stain until fluorescent signals of single fluorophore-bound secondary antibodies can be detected. The same titration process is performed with a primary antibody to determine the saturation of epitopes on the targeted protein. The data from the primary and secondary titration experiments is then used to estimate the number of secondary antibodies that bind to each primary. Taken together, this information allows for estimations of protein copy number in experimental conditions by comparing the density profile of localizations in an experimental cluster to the profile of a known single protein.

#### Other calibration tools.

Alternative methods for deducing protein copy number include those that utilize a fluorescently labeled calibration tool with a known copy number against which experimental clusters can be compared. Some recent examples include DNA origami, bacterial homo-oligomers, and nuclear pore complex (NPC) proteins (25, 44, 170). For example, DNA origami structures can be designed with extreme precision in dimension (several nanometers) and can be made to support regularly spaced handles that can be conjugated with multiple copies of a fluorescently labeled protein of interest (71, 139). Since the copy number of proteins attached to the origami is already known, it can be used as a calibration standard, allowing it to account for the photophysical and labeling variables mentioned above. The copy number of the protein of interest in the experimental sample can then be determined by comparing the SMLM localization distributions of the sample to calibration distributions calculated for the DNA origami standard (25).

One major drawback of both the DNA origami approach and the bacterial homo-oligomer is that the copy number of the proteins within the calibration standard is typically low (1–6 proteins), and extrapolating the calibration to larger protein complexes gives large errors in copy number estimation (25). To overcome this problem, well-characterized subunits of the NPC offer another option for protein counting in clustered SMLM data (170). Nup96, for instance, forms ring structures of 16 subunits on both the cytoplasmic and nucleoplasmic faces of the NPC for a total copy number of 32 (178). However, the NPC offers a fixed stoichiometry for quantification, and it is not clear how well the calibration will perform when the protein copy number of interest is much smaller or larger than the Nup96 stoichiometry (77). Thus, there is still a need for flexible, easy-to-use calibration standards whose copy number can easily be tuned over a large range.

### Colocalization

Multicolor imaging has been a powerful tool for revealing the spatial relationships among different molecular species and for understanding their interdependence. Methods that can quantify the spatial relationship and colocalization in standard microscopy images have been around for a long time and typically fall into two categories: pixel-based methods and object-based methods. The former includes the Pearson’s correlation, Manders overlap, and Spearman correlation coefficients. Object-based methods typically rely on segmentation of individual objects or structures and measurement of their overlap percentage or nearest-neighbor distances. Both types of analysis have been adapted and applied to super-resolution microscopy. In the simplest scenario, super-resolution images can be rendered as pixel-based images, and the typical correlation coefficients can be computed on the pixel-based data (164). However, this approach has the drawback of arbitrary choice of pixel size and potential pixelation artifacts. Pair-correlation and other statistical analysis (see the section titled Statistical Analysis of Single-Molecule Localization Microscopy Data) can also be directly applied to point localizations to quantify the (average) spatial relationship between different molecular species in SMLM data (101, 138, 153, 155, 166, 187). Colocalization analysis can also be applied in conjunction with clustering and segmentation algorithms (4, 95, 126). For example, Voronoi tessellation has been extended to quantify the overlap of segmented clusters in multicolor SMLM images in 2D (4) and 3D (95). Coloc-Tesseler computes the normalized pair-density from the overlapping Voronoi diagrams of two molecular species to quantify their spatial colocalization in a density- and parameter-free manner. In another approach, clusters segmented by DBSCAN or Voronoi in one channel are assigned as reference clusters, and the clusters of the second channel are compared to these. A cluster is then considered to be colocalized with a reference cluster if the proportion of its localizations that fall within this reference cluster is higher than a predetermined threshold (60).

## ADVANCED QUANTITATIVE METHODS

In the sections below, we describe more advanced quantitative tools for super-resolution microscopy. This list is by no means exhaustive, but it offers details on the most commonly used methodologies.

### Structural Biology and Structural Modeling

While super-resolution light microscopy has great potential for determining the structure of multiprotein complexes in situ (98, 161), several limitations make it challenging to directly achieve the structural-level resolution that is typical in cryo-electron microscopy (EM) and cryo-electron tomography (ET). First, the spatial resolution of super-resolution light microscopy remains at the nanometer scale, in contrast to the Ångström-scale resolution provided by cryo-EM and cryo-ET. In addition, while fluorescence tagging provides high contrast and molecular specificity, it only allows visualization of a few proteins at a time. This makes visualization of all subunits within large molecular complexes difficult. Additionally, in super-resolution light microscopy, the subunit of interest is rarely directly visualized. Instead, a label that can be separated from the subunit by tens of nanometers is visualized (92). Still, concepts that are typically used in single-particle cryo-EM have been adapted for super-resolution microscopy in select examples to determine the subunit arrangement of multiprotein complexes (116, 141, 160, 169). In this case, a subunit within a specific molecular structure is labeled and visualized with super-resolution microscopy. Many such images are segmented, aligned, and averaged together to determine the position of the subunit with nanometer precision. For a symmetric structure, like the NPC, this procedure can be repeated individually for multiple subunits to determine their relative position within the NPC complex in 2D (100, 169) as well as in 3D (141). For structures that are not symmetric, a reference subunit can be imaged together with the subunit of interest in multicolor to enable image alignment and averaging based on the reference image (116). This approach was used to produce a pseudotemporal order of molecular events that take place during clathrin-mediated endocytosis (116). Moreover, when applied to purified proteins, 2D images of single-protein subunits of mixed orientation can be used to reconstruct a 3D volumetric image using established EM analysis routines (160).

All of these approaches require robust data analysis workflows for segmenting, classifying, aligning, and averaging structures of interest. Segmentation is typically achieved through methods like DBSCAN or Voronoi. For alignment and averaging, different approaches have been developed (98). In one class of methods, a high-resolution, pixel-based representation of the point cloud data is reconstructed by binning the localizations into intensity-based pixels. Image cross-correlation can then be used to align and average the images (141, 169). However, approaches that rely on reconstructed images can be prone to reconstruction artifacts, and thus, direct analysis of the point cloud data may be preferable. Methods that work on the point cloud data include template-based methods that use a priori knowledge of the structure of interest (21, 100), as well as template-free methods that generate a data-driven template by maximizing a merit function from the 2D or 3D localizations (65, 66, 153). Finally, after averaging, simple geometric expressions are used to model the biological structures (e.g., circles, Gaussian profiles, lines) to get access to subunit information.

### Single-Particle Tracking

In SPT (Figure 2), molecules are tracked in time to quantify dynamic behavior in 2D and 3D in living cells (72, 104, 129). SPT in its most standard version requires sparse labeling of the protein of interest such that the molecules do not overlap in a diffraction-limited volume. However, SPT can be combined with PALM (104, 147) to increase the density of molecules that are imaged and tracked. SPT has also more recently been combined with MINFLUX microscopy (11, 148) to improve localization precision and temporal resolution. The analysis of SPT data shares many commonalities with SMLM analysis, including probe detection and localization, but with the addition of linking the localized points from one frame to the next to create trajectories. Many software packages are publicly available, including multiple-target tracing (MTT) (156), u-track (79), TrackMate (43, 171), and Single-Molecule Analysis by Unsupervised Gibbs sampling (SMAUG) (84), to help with the analysis of different types of SPT experiments.

The first step in SPT analysis is the probe localization. This is a more complicated process than the localization of typical SMLM data, as the probe PSFs can be deformed due to movement (i.e., motion blur effect) (36) and as high temporal resolution is needed to accurately follow the dynamics (121); both of these factors lower the localization precision. The use of MINFLUX makes this step more robust, as it provides very high localization precision without the need for high photon counts (approximately 1 nm precision), which allows for faster dynamics to be investigated (100-fold enhancement) (11). Additionally, methodological advances have also been made to robustly identify single particles in noisy situations (109).

The second step of SPT analysis is linking the detected probes in subsequent frames to form trajectories in time. Even though the concept is simple, many problems arise in this step, such as crossing trajectories or gaps due to blinking (for more information, see 105), but these issues can be minimized using experimental optimization methods such as implementing strategies for reduced blinking (34), sparse labeling, or simultaneous multicolor SPT (23). Other efforts have been made on the computational side with the use of deep learning algorithms (43, 51) or Bayesian statistics (26, 129).

The final step is to interpret the linked trajectories and use them for quantification with one of the many tools available. The extracted information can be classified according to two main approaches. The first class, Lagrangian methods, focuses on quantifying individual molecule dynamics. In this class, aspects such as diffusive motion (e.g., mean square displacement, apparent diffusion coefficient) or transient state kinetics are characterized (51, 72, 104). The second class of approaches is made up of the Eulerian methods, which focus on characterizing distinct regions in the sample by looking at the dynamics of the molecules passing through (14, 108, 156). Both types of methods have their pros and cons, but generally speaking, Lagrangian methods are computationally expensive in most applications due to the fact that they track all individual particles (although this is a drawback in most SPT calculations and does not affect tracking performance). However, due to this tracking, Lagrangian methods give access to information on every individual particle. In contrast, the Eulerian methods are faster, as they calculate ensemble statistics, with the drawback that subtle differences between individual particles will not be noticed. SPT has been applied in a diverse set of biological contexts, including dynamics of membrane receptors (104, 108, 156), actin dynamics (2, 50, 189), intracellular transport dynamics (127, 129, 191), and transcription factor mobility in the nucleus (78, 93, 167, 188).

### Stimulated Emission Depletion-Fluorescence Correlation Spectroscopy

FCS is a quantitative spectroscopic method for measuring molecular interactions, determining molecular concentrations, and observing the dynamic movement of molecules in living cells. This method relies on analyzing temporal fluctuations in the fluorescent intensity of small quantities of labeled molecules as they pass through a spot of focused light (focal volume) (85). Early use of FCS was limited by diffraction to focal volumes of approximately 250 × 250 × 500 nm^3^, and averaging over these large volumes of molecules made it very challenging to visualize heterogeneities caused by molecular interactions that take place at length scales smaller than the diffraction limit.

As STED intrinsically uses a confocal geometry, it is highly compatible with FCS measurements. STED-FCS shrinks the focal volume to subdiffraction dimensions, allowing the observation of molecular dynamics that happen at small length scales, thus extending the spatiotemporal resolution of light microscopy. Since its introduction, STED-FCS has been applied to studies of lipid and protein diffusion in the plasma membranes of eukaryotic cells and adapted for scanning detection of multiple areas for the observation of heterogeneity in membrane architecture (69, 151, 157).

### Biosensing

Determining the biochemical activity of cellular constituents is a core goal of cell biology, and this field was revolutionized by genetically encoded sensors, which typically consist of two different domains: the sensing domain, which is responsible for the sensing of presence or absence of activity, analyte, or interaction, and the reporting domain, which produces the measurable signal. The two main classes of these sensors are FRET-based sensors (two fluorophores) and biosensors that use the change in fluorescence intensity as a read-out (single fluorophore). The FRET-based sensors can provide absolute quantification of the activity but have several drawbacks related to their large construct size and their limited multiplexing capabilities. Single-fluorophore biosensors, in contrast, are fast and small and can be used in multiplexed experiments, but as their emission depends on the local concentration of the probe, absolute numbers remain difficult to obtain (with some exceptions; see 16), and response saturation cannot be detected. Nevertheless, biological activity has been quantified and monitored in real time with different microscopy modalities including super-resolution optical fluctuation imaging. Examples of applications include calcium sensing (22, 46), pH sensing (96, 133), and voltage sensing (49, 83). Greenwald et al. (52) provide a more complete overview.

### Deep Learning

Recently, the use of deep learning in super-resolution microscopy has experienced an immense increase in popularity. The main applications are emitter or object detection (117, 132), object classification (63, 99), tracking (7, 51), segmentation (68, 168), preprocessing (90, 113, 185), and augmented microscopy (123, 180). In their simplest form, super-resolution raw images can be described as a convolution of the PSF with a point-like emitter with the addition of background and noise. Given that convolutional neural networks use convolutions as sliding filters of the input features, their usefulness in this application is evident. As a result, deep learning methods remain robust in situations of low signal-to-noise ratio, at higher emitter densities, and with incomplete labeling (20, 47, 124, 132) and will typically outperform classical image processing techniques when they are evaluated in terms of robustness, precision, accuracy, and fidelity. Regardless of their indispensable role and future potential in quantitative super-resolution microscopy, there are several drawbacks to be considered, including their need for powerful resources, their high entry threshold for users, a large degree of parameter tuning during training, and their limited interpretability. Fortunately, the community has recognized these limitations and is actively seeking to resolve them with, for example, the initiation of the ZeroCostDL4Mic platform (179), an open-source resource with tutorials, Jupyter notebook availabilities, and many different example data sets.

### Classification

Classification (Figure 2) is important when discriminating multiple types of known objects from one another (8, 32) or when detecting new groups present in a data set (60, 141). Classification can be performed in two different ways. The first is by use of an unsupervised procedure [e.g., hierarchical cluster analysis (HCA), topological data analysis], which requires no user input and is data driven but is usually less precise in its quantification. The second type of classification algorithm is made up of supervised procedures (e.g., deep learning–based methods, support vector machine) where a ground truth must be provided to the algorithm during the training step so that the classification model can learn the features that set the different classes apart. In both cases, the classification is done using different variables that describe the structures adequately. These descriptors can be extracted from the data or reconstructed images and can differ greatly from application to application. For example, Gyparaki et al. (60) used an iterative HCA on eight features that describe super-resolved Tau protein aggregates (e.g., number of localizations, aggregate area, aspect ratio) to find 22 different classes that may represent different stages in Tau aggregation. Other studies (32, 141) have used classification in combination with structural modeling to extract relevant biological or structural parameters.

## CONCLUSION AND FUTURE PERSPECTIVES

Since the introduction of super-resolution microscopy techniques to biological investigations, the field has grown and innovated in many exciting ways. The emphasis of early studies was primarily on the development of new techniques for visualization. Now that these techniques are well-established, attention can and has already been directed toward quantifying the information acquired through them to gain new insights into the underlying biology. An important aspect of quantitative super-resolution imaging is robust preprocessing of the images (e.g., background removal, localization), and many efforts have been made to develop quantitative tools that maximize precision, accuracy, fidelity, etc., efforts that subsequently have positive effects on the postprocessing quantification. What many of the quantification methods have in common is that extensive knowledge of the (physical) basis of the data or images (i.e., a priori knowledge) serves as a basis to maximize the information that can be quantified. However, each method has its own advantages and drawbacks, as discussed throughout this work. Ultimately, the method of choice is highly dependent on the biological application and the biological questions being addressed.

As inspiring and encouraging as the topics reviewed are for applications in cell biology and beyond, challenges remain. Many of the methods covered were developed by independent research groups that use different programming environments, resulting in analysis pipelines that are not necessarily streamlined and inevitable technological compatibility issues. To address this problem, the creation of an open-source tool that combines these powerful algorithms to standardize and generalize these platforms should be the subject of a greater community effort. Moreover, it would be appropriate to include an active feedback system in this pipeline that tells users at each step of the analysis how reliable and robust a procedure is and how it influences the quality of the data and results. This will lower the threshold for nonexpert users to use super-resolution microscopy in their research (as it is complicated to navigate the vast range of available methods) and will further improve reliability and reproducibility of results by removing user dependencies.

Exciting opportunities have also arisen with the recent developments in multiplexed microscopy, as well as MINSTED and MINFLUX, as these techniques allow for imaging a large number of molecular species or achieving much higher localization precisions than what is typically achieved with SMLM [1–2 nm, or even in the Ångström range (184)]. These higher-resolution techniques hold great promise for probing spatiotemporal dynamics of molecules that were previously inaccessible. Moreover, this unprecedented precision will further incentivize studies and applications in structural biology, as it allows access to the subunits of the biological structures without the need for averaging. These advancements bring super-resolution microscopy closer to cryo-EM or cryo-ET, with the added benefit of live-cell compatibility, and allow for studying cell biology in native conditions.

Finally, deep learning algorithms have accelerated advances in the field by taking advantage of the intrinsic structure of fluorescence images, but the rise of algorithms based on deep learning has certainly not hit its peak, and these algorithms still have a lot of unused potential in this field. Developments from Ounkomol et al. (123) and Wang et al. (182), for example, enable super-resolution modalities without the need for expensive equipment by using deep learning to predict fluorescence images from transmitted-light images. This so-called augmented microscopy is promising, but more advances are needed before this technology can be used in quantitative experiments. A promising candidate to achieve this goal is the use of generative adversarial networks (GANs), an up-and-coming deep learning technique increasingly being applied to super-resolution microscopy (180, 190). Their structure allows for automatic pattern discovery in the data, which is then used to generate new data that cannot be distinguished from experimental data. They are especially powerful in applications where both the input and output of the deep learning algorithm are images (i.e., image-to-image translation) and may play a key role in democratizing quantitative super-resolution modalities. Some of the drawbacks of existing deep learning algorithms (e.g., their bias toward training data) may also potentially be overcome using GANs, as they are excellent at generating realistic examples across a range of problems. Moreover, to date, deep learning algorithms have been black box techniques (information is known only about the input and output). Gaining insight into the image features that are important for successfully completing the algorithm’s task is a line of research that should be further explored, as the understanding of what makes an algorithm successful will lead to more robust and precise results, given that this information can be exploited throughout the optimization.

From the topics discussed in this review, it is clear that progress in this field will undoubtedly continue, and exciting new discoveries will be made based on the quantitative analysis of the molecular mechanisms that drive living systems.
